# Steatosis Grade is the Most Important Risk Factor for Development of Endothelial Dysfunction in NAFLD

**DOI:** 10.1097/MD.0000000000003280

**Published:** 2016-04-08

**Authors:** Ferdane Sapmaz, Metin Uzman, Sebahat Basyigit, Selcuk Ozkan, Bunyamin Yavuz, Abdullah Yeniova, Ayse Kefeli, Zeliha Asilturk, Yasar Nazligül

**Affiliations:** From the Keçiören Education and Training Hospital (FS, MU, SB, AY, AK, ZA, YN), Gastroenterology Department; and Keçiören Education and Training Hospital (SO, BY), Cardiology Department, Ankara, Turkey.

## Abstract

It is shown that there are strong associations between nonalcoholic fatty liver disease (NAFLD) and endothelial dysfunction. The aim of our study was to reveal whether steatosis or fibrosis score is more important in the development of endothelial dysfunction in patients with NAFLD in a prospective manner.

This cross-sectional study included 266 subjects. These subjects were divided into 2 groups depending on presence of hepatosteatosis sonographically. Patients with hepatosteatosis were also divided into 3 subgroups depending on degree of steatosis: grade 1, 2, and 3. In all patients, Aspartate aminotransferase-to-Platelet Ratio Index and Fibrosis-4 (FIB4) scores were calculated. In addition, flow-mediated dilatation (FMD) measurements were recorded.

There was NAFLD in 176 (66.2%) of 266 patients included. There were no significant differences in sex and age distributions between patients with NAFLD (group 1) and controls without NAFLD (group 2) (*P* = 0.05). Mean Aspartate aminotransferase-to-Platelet Ratio Index score was significantly higher in group 1 compared with the control group (*P* = 0.001), whereas no significant difference was detected regarding FIB4 scores between groups (*P* = 0.4). Mean FMD value was found to be significantly lower in group 1 (*P* = 0.008). Patients with grade 3 hepatosteatosis had significantly lower FMD values than those with grade 1 steatosis and controls (*P* = 0.001). In univariate and multivariate analyses in group 1, no significant difference was detected regarding mean FMD measurements (*P* = 0.03). Again, no significant difference was detected in mean FMD measurement between FIB4 subgroups among patients with NAFLD and the whole study group (*P* = 0.09).

The endothelial dysfunction is associated with steatosis in patients with NAFLD.

## INTRODUCTION

Nonalcoholic fatty liver disease (NAFLD) is defined as a lipid amount greater than 5% to 10% of liver weight or lipid vacuoles filling more than 5% of hepatocytes in histopathological examination of individuals with alcohol consumption at a level that is not thought to be harmful for liver.

Nonalcoholic fatty liver disease is the most common liver disorder in developed countries.^1^ A recent study using the National Health and Nutrition Examination Survey (NHANES) found a 30% prevalence of NAFLD in the United States between 2011 and 2012.^2^ The main causes of NAFLD are associated with insulin resistance, metabolic syndrome, and serious lipid metabolism disorders.^3^ NAFLD represents a spectrum of liver conditions ranging from simple steatosis to steatohepatitis, fibrosis, and, ultimately, cirrhosis.^4^ Biopsy confirms the histologic presence of hepatic steatosis and fibrosis is the diagnostic reference standard for NAFLD; however, it is an invasive procedure.^5^ Therefore, noninvasive, safer staging systems have been developed in NAFLD. Several such fibrosis scores have been developed and validated in large studies on adults with NAFLD.^6^ In our clinical practice, we mostly use Aspartate aminotransferase (AST)-to-Platelet Ratio Index (APRI) and Fibrosis-4 (FIB4) score.

A strong association between NAFLD and cardiovascular disease has been long suspected, and recent studies have confirmed that cardiovascular disease is the single most important cause of mortality in this patient population.^7^ Thus, the early identification and management of these cardiovascular risks should help reduce NAFLD-related complications.

In vascular diseases, endothelial dysfunction is a systemic pathological state of the endothelium, which can be broadly defined as an imbalance between vasodilating and vasoconstricting substances produced by the endothelium.^8^ Impaired endothelial function occurs during the early course of atherosclerosis.^9^ Brachial artery flow-mediated dilation (FMD) is the most frequently utilized noninvasive test for assessing endothelial function as the result of endothelial release of nitric oxide.^10^ Several studies have shown that patients with NAFLD were significantly associated with endothelial dysfunction.^11–13^

The aim of our study is to reveal, whether the steatosis score or the fibrosis score is more important in the development of endothelial dysfunction in patients with NAFLD in a prospective manner.

## METHODS

### Study Population

This is a prospective, cross-sectional study investigating NAFLD and hepatic fibrosis as a risk factor for endothelial dysfunction. This study was conducted by using a registry of participants, who were referred to the outpatient clinic of the Division of Gastroenterology for the determination of hepatic function between March 2014 and July 2015, at a single center in Turkey. This study was approved by the Institutional Ethical Board and is in accordance with the Helsinki Declaration. The written informed consent about the study and a standard questionnaire regarding their personal medical history, present medications, family history, and life style habits were obtained from all participants. Physical examinations, laboratory assays, and imaging studies were performed after a fasting period of at least 12 hours.

During the study period, we examined 495 patients. We excluded subjects with a history of chronic alcohol consumption (n = 18), chronic liver disease (n = 12), and seropositivity of hepatitis B virus (n = 54) and hepatitis C virus (n = 20). We also excluded subjects who had a history of cardiovascular disease (n = 67), cerebrovascular disease (n = 25), and peripheral vascular disease (n = 19). After exclusion of these subjects, 280 patients were eligible for the study. A total of 266 patients were included in the study. Participation rate was 95%.

We divided the patients into 2 groups according to their hepatic ultrasonographic findings: those with normal hepatic ultrasonography (USG) and those with NAFLD. Patients with NAFLD were also divided into 3 subgroups: stage 1 hepatosteatosis, stage 2 hepatosteatosis, and stage 3 hepatosteatosis.^14^

### Measurements, Definitions, and Laboratory Assays

Anthropometric measurements, blood pressure, and laboratory tests were measured after a 12-hour fasting period. Trained nurses measured the height and weight of the participants. Blood pressure was measured after a 5-minute rest with a standard mercury sphygmomanometer. The presence of hypertension was defined according to the 2013 hypertension guidelines of the European Society of Hypertension and the European Society of Cardiology,^15^ or as the use of antihypertensive medication. Waist circumference was measured at the umbilicus level. Increased waist circumference was based on the definition of the Regional Office for the Western Pacific Region of World Health Organization criteria.^16^ The body mass index was calculated as kg/m^2^. Diabetes mellitus was determined by American Diabetes Association 2003 guidelines.^17^ Metabolic syndrome was defined as having at least 3 of the criteria set by the Adult Treatment Panel III criteria, as updated by the American Heart Association.^18^

A venous blood sample was drawn from an ante-cubital vein. Liver enzymes, lipids, glucose, and other biochemical markers were measured in the sera of subjects. The Homeostasis Model Assessment for Insulin Resistance (HOMA-IR), an index of IR, was calculated with the serum insulin and glucose values of the individuals.^19^ Presence of IR was defined as having a HOMA-IR score ≥2.5.

### Evaluation of Hepatosteatosis

Hepatic USG scans (a 3.5-MHz transducer [Logiq P5; GE]) were performed for all participants by 2 trained gastroenterologist, blindly and independently. The diagnosis of NAFLD was made on the basis of 4 known criteria, namely, hepatorenal echogenic contrast, liver brightness, deep attenuation, and vascular blurring.^20^

### APRI and FIB4 Scores

The APRI was calculated as AST/upper limit of normal (ULN)/platelets × 100.^21^ The FIB4 score was calculated using the following formula: FIB4 = (age × AST)/(platelet count [10.9/L] ×  
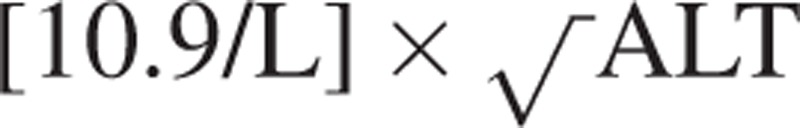
ALT [alanine aminotransferase]).^22^

### Interpretation

#### APRI Scores

In a meta-analysis of 40 studies, investigators concluded that an APRI score greater than 1.0 had a sensitivity of 76% and specificity of 72% for predicting cirrhosis. In addition, they concluded that APRI score greater than 0.7 had a sensitivity of 77% and specificity of 72% for predicting significant hepatic fibrosis.^21^

#### FIB4 Scores

Using a lower cut-off value of 1.45, a FIB4 score <1.45 had a negative predictive value of 90% for advanced fibrosis (Ishak fibrosis score 4–6, which includes early bridging fibrosis to cirrhosis). In contrast, a FIB4 score >3.25 would have a 97% specificity and a positive predictive value of 65% for advanced fibrosis. In the patient cohort in which this formula was first validated, at least 70% patients had values <1.45 or >3.25. Authors argued that these individuals could potentially have avoided liver biopsy with an overall accuracy of 86%.^22^

### Measurements of Flow-mediated Dilatation and Flow of Brachial Artery

We measured FMD of the brachial artery according to the International Brachial Artery Reactivity Task Force guidelines^23^ using a novel ultrasound system equipped with an edge-tracking system for 2-dimensional imaging and a pulsed Doppler flow velocimeter for automatic measurement. The right brachial artery was scanned over a longitudinal section, 3 to 5 cm above the right elbow and the arm was kept in the same position throughout the study. A pneumatic tourniquet was placed around the distal forearm. First, the diameter of the brachial artery was recorded in the cubital region at rest. Subsequently, the cuff was inflated to 50 mm Hg above the systolic blood pressure of patients for 5 minutes and then increased flow was induced by sudden cuff deflation. The diameter of the artery was monitored continuously at the same point and the maximum dilatation after deflation was recorded. The diameter of the brachial artery was measured from the anterior to the posterior interface between the media and adventitia (“m line”) at a fixed distance. All measurements were made at both end diastole and end systole to avoid possible errors resulting from variable arterial compliance. The change in diameter caused by FMD was expressed as the percentage relative to the diameter in the initial resting scan. Cut-off value for decreased FMD were determined as <10%.

### Statistical Analysis

The statistical analysis was performed with The Statistical Package for the Social Sciences version 15.0 (SPSS, Chicago, IL). The statistical results are presented as the mean ± standard deviation/standard error, percentages, or median (minimum-maximum). We used 1-way analysis of variance test for continuous variables. Risk estimation and comparison of categorical data were made by the chi-square test. Odds ratio (OR) is presented together with its 95% confidence interval (CI). Effect of variables on dependent measurement were analyzed with linear regression analysis. Multivariate linear regression analysis was used to compare dependent variables between groups. The correlations between NAFLD scores and noninvasive fibrosis scores were investigated by Kendall tau correlation test. The degree of agreement between the scores was measured with the Kendall tau-b correlation coefficient as for ordinal-level variables. Values less than 0.2 are associated with very poor agreement, 0.2 to 0.40 with slight agreement, 0.4 to 0.6 with moderate agreement, 0.6 to 0.8 with substantial (good, high) agreement, and values greater than 0.8 with excellent (almost perfect) agreement.^24^ The concordance between two USG measurements was also measured with Mc Nemar test. *P* value <0.05 was considered statistically significant.

## RESULTS

### Baseline Characteristics of the Subjects

A total of 495 subjects were eligible. Of these, 266 (95% participation rate) were included in the final analysis, after excluding 229 subjects according to exclusion criteria. The mean age of the enrolled subjects was 50.3 ± 13.2 years (range 19–82 years), and 109 of them were male (41%). A total of 176 subjects (66.2%) had NAFLD. Results of USG measurements by 2 experts have good concordance (Mc Nemar test, chi-square value: 0.109, *P* = 0.32).

The demographic and clinical characteristics of the study groups are summarized in Table 1.

**TABLE 1:**
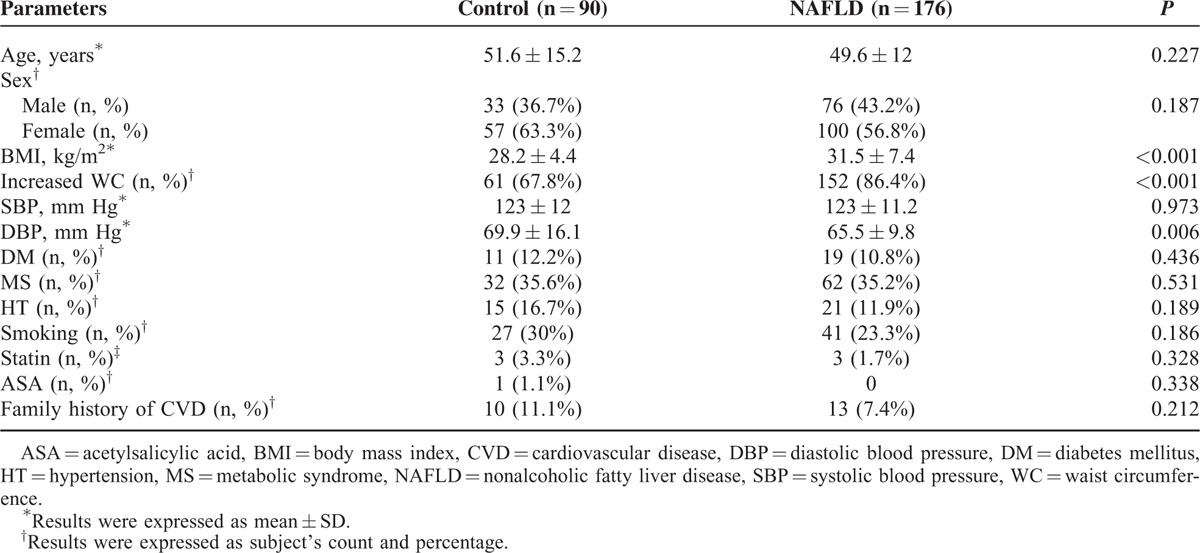
Demographic and Clinical Characteristics of Study Groups

There was no significant difference in the distribution of age and sex among the groups. NAFLD was strongly associated with central obesity and was significantly related to higher body mass index values (*P* < 0.001) and waist circumferences (*P* < 0.001).

### NAFLD, Fibrosis, and FMD

Table 2 reports biochemical characteristics of patients with and without NAFLD.

**TABLE 2:**
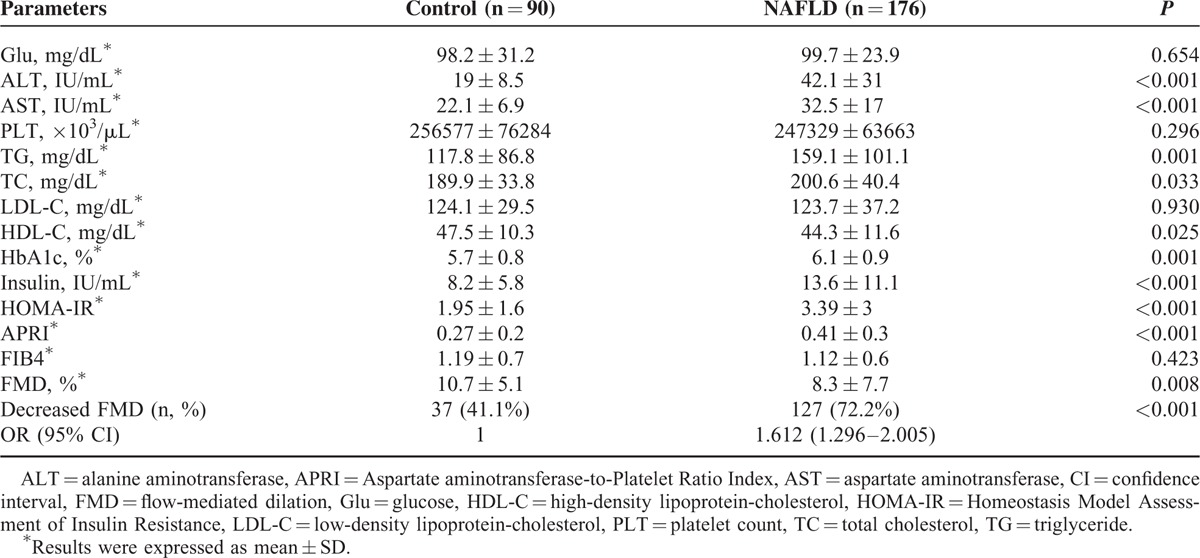
Laboratory Findings of Study Groups

Patients with NAFLD had higher levels of alanine aminotransferase (ALT) (*P* < 0.001), AST (*P* < 0.001), HOMA-IR (*P* < 0.001), total cholesterol (*P* = 0.003), triglyceride (*P* = 0.001), insulin (*P* < 0.001), HbA1c (*P* = 0.001), APRI score (*P* < 0.001), and had lower levels of high-density lipoprotein-cholesterol (HDL-C) (*P* = 0.025) and FMD (*P* = 0.008). There was no significant difference in FIB4 score between the 2 groups.

In the NAFLD group, the ratio of patients with a lower FMD (<10%) value was higher. The presence of NAFLD was a risk factor for decreased FMD (OR 1612, *P* < 0.001).

Due to the clinical and demographic differences between groups and the presence of factors that impact FMD, linear regression analysis was performed. Regression analysis revealed that NAFLD score and smoking have independent effects on FMD (respectively, *P* = 0.003, *P* = 0.001) (Table 3).

**TABLE 3:**
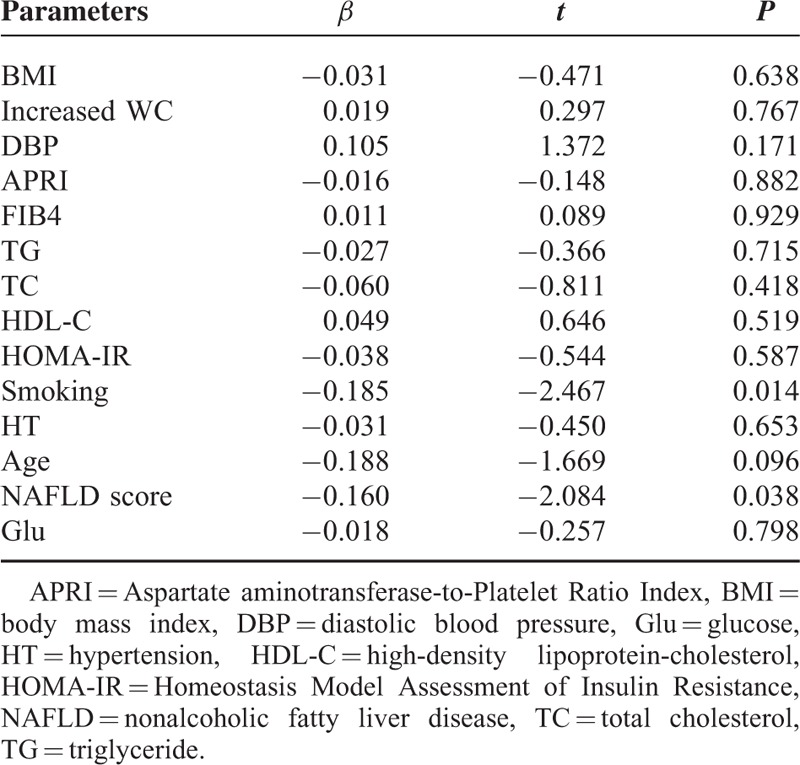
Linear Regression Analysis of Factors Affecting Flow-mediated Dilation

The comparison between FMD measurements and NAFLD, APRI, and FIB4 subgroup analysis are shown in Table 4.

**TABLE 4:**
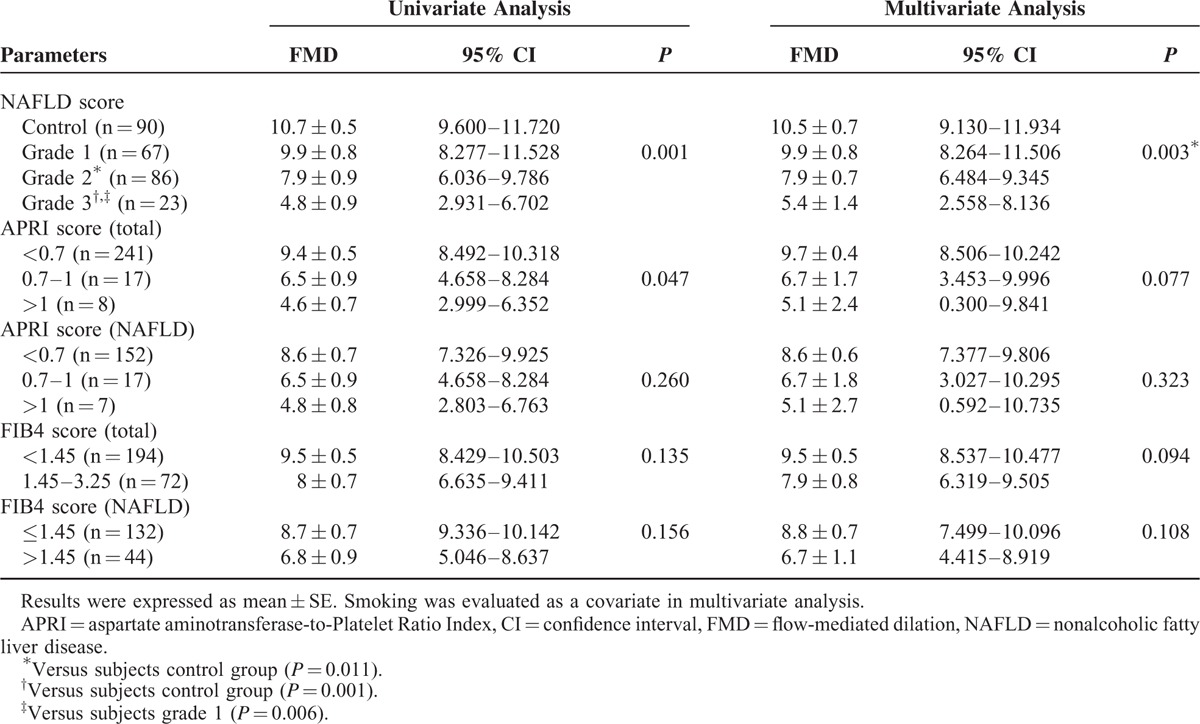
Comparison of FMD Measurements According to NAFLD and Fibrosis Scores

According to both univariate and multivariate analyses and also to covariate results, patients with grade 3 hepatosteatosis have lower FMD values compared with both patients with grade 1 hepatosteatosis and the group in which NAFLD is absent (respectively, *P* = 0.001, *P* = 0.003).

**FIGURE 1:**
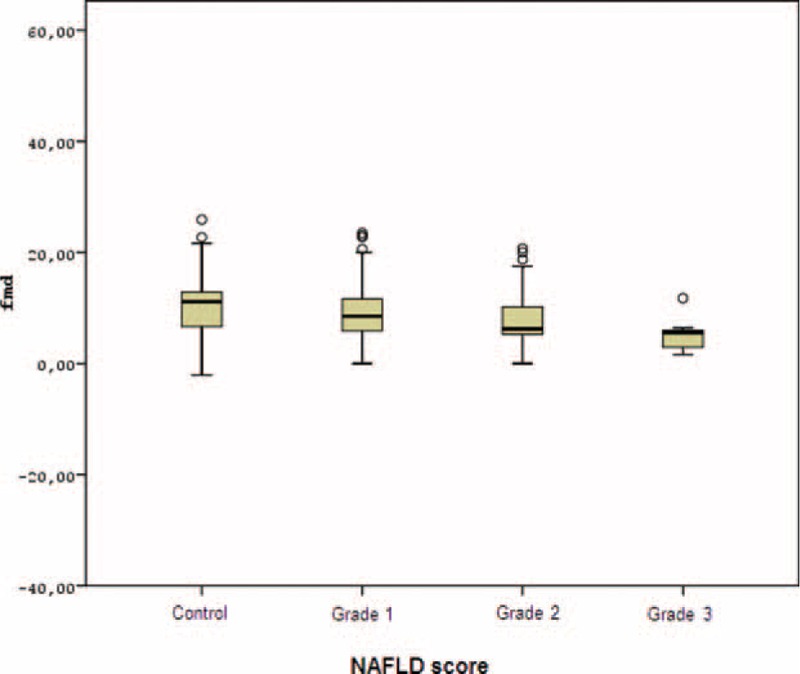
Comparison of FMD measurements according to NAFLD and fibrosis scores.FMD = flow-mediated dilatation, NAFLD = nonalcoholic fatty liver disease.

In patients with grade 2 hepatosteatosis, FMD values were significantly lower compared with the control group (*P* = 0.001) (Figure 1).

A comparison of the FMD measurements between APRI subgroups were analyzed separately for NAFLD patients in all study groups. Although there were statistically significant differences between subgroups in the univariate analysis in terms of average FMD measurements, these statistically significant differences could not be shown in the multivariate analysis.

A comparison of average FMD measurements between FIB4 subgroups was analyzed separately, both within the entire group and in patients with NAFLD. All patients were divided into 2 groups according to their FMD measurements: FMD measurement ≤1.45 and FMD measurement >1.45. In univariate and multivariate analyses, there were no significant differences between FIB4 subgroups in terms of FMD measurements, both within the entire group and in patients with NAFLD.

The correlation between NAFLD scores measured by USG and noninvasively measured fibrosis scores were evaluated with Kendall tau-b test. The agreements between APRI and NAFLD scores and between NAFLD and FIB4 scores were shown to be very poor (Kendall tau-b: 0.122) (Table 5).

**TABLE 5:**
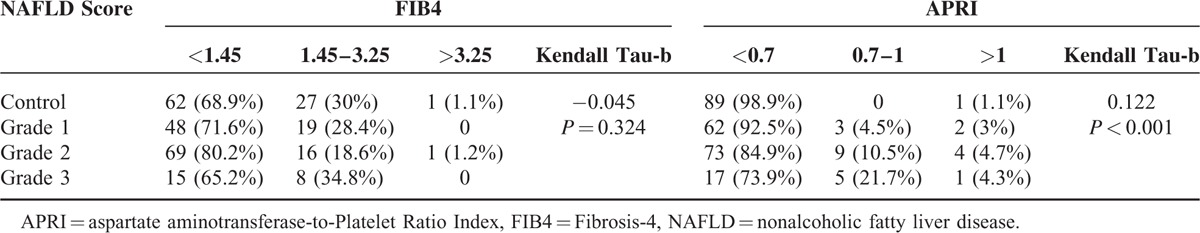
Concordance Between Ultrasonographic NAFLD Scores and Noninvasive Fibrosis Scores

## DISCUSSION

To the best of our knowledge, this is the first available published study on the association between fibrosis scores in NAFLD and endothelial dysfunction.

Nonalcoholic fatty liver disease is a disease spectrum ranging from simple steatosis to nonalcoholic steatohepatitis (NASH), fibrosis, and ultimately cirrhosis.^25^ In the published data, mortality rates from coronary heart disease in patients with NAFLD were equal to those related to cirrhosis.

Nonalcoholic fatty liver disease is also strongly associated with risk factors for atherosclerosis, such as obesity, dyslipidemia, hypertension, type 2 diabetes mellitus, and insulin resistance.^26^ NAFLD is now considered to be a hepatic manifestation of metabolic syndrome.

Similarly, in our study, NAFLD was strongly associated with central obesity and significantly related to higher body mass index values (*P* < 0.001).

Endothelial dysfunction is an important process accepted as a predictor of atherosclerosis.^27^ Several clinical factors that play a major role are common in etiologies of both endothelial dysfunction and NAFLD, such as obesity, diabetes mellitus, dyslipidemia, and metabolic syndrome.

There are different methods that can evaluate endothelial function except FMD. These methods can be listed as follows: brachial artery measures—flow-mediated dilatation (%), hyperemic mean flow velocity (cm/s); peripheral arterial tonometry measures—peripheral arterial tone ratio; arterial tonometry measures—1000/carotid-femoral pulse wave velocity (ms/mm), forward-wave amplitude (mm Hg), mean arterial pressure (mm Hg).

Flow-mediated dilatation is an ultrasound-based method in which arterial diameter is measured in response to an increase in shear stress, which causes endothelium-dependent dilatation.^28^ Endothelial function assessed by this method correlates with invasive testing of coronary endothelial function, and also with the severity and extent of coronary atherosclerosis.

In our study, FMD was found to be markedly reduced in patients with NAFLD compared with the control group. Another important finding was that this decrease was even more prominent in patients with grade 3 NAFLD. Recently, it has been shown that NAFLD grade is a strong risk factor for endothelial dysfunction that was grossly determined by reduced FMD.

It is very likely that the different mechanisms involved in the pathogenesis of endothelial dysfunction in patients with NAFLD have a varying relevance to individual genetic background. Possible mechanisms linking NAFLD with impaired endothelial function may be subclinical inflammation, which is implicated in the pathophysiology of NAFLD. A possible mechanism linking NAFLD with endothelial dysfunction may be insulin resistance.^29^ Insulin resistance is associated with excessive ectopic fat accumulation and low-grade systemic inflammation. Moreover, neither elevated free fatty acid nor liver injury itself may contribute to systemic inflammatory process and oxidative stress.^30^

The relationship between NAFLD and endothelial dysfunction has been shown in many studies. In 2005, Villanova et al^31^ reported that FMD was significantly reduced in NAFLD population. In this case-control study, NAFLD, diagnosed by liver biopsy or by ultrasound, predicted a reduced FMD after adjusting for age, sex, BMI, and insulin resistance. Another study reported that endothelial dysfunction was worse in NASH compared with simple steatosis and control group, suggesting that the inflammation in the liver has a role.^32^

Although we found that NAFLD is strongly associated with decreased FMD and the degree of NAFLD is correlated with FMD, we did not found any association between FMD measurements and noninvasive fibrosis indices. In addition, linear regression analysis showed that fibrosis index had no effect on FMD results. In the literature, there has not been any data on whether hepatic fibrosis effects endothelial dysfunction or not. Our study is the first which evaluates the relationship between endothelial dysfunction and hepatic fibrosis. However, number of patients which had APRI index >1 is limited in the present study. This condition makes difficult to conclude exactly that hepatic fibrosis is not important in the development of endothelial dysfunction. However, this study is pioneer for further studies, and different pathophysiologic mechanisms involved in fibrosis development in NAFLD course may explain this result.

It is important to note that our study had some limitations. The first is relatively small sample size. Secondly, serum levels of endothelial markers, which also involve in the development of fibrosis and measurements of fibro-scan, were not included. Lastly, liver biopsy was not implemented because it is an invasive procedure. Further studies are needed without these limitations.

## CONCLUSIONS

In conclusion, this is the first study which revealed the relationship between endothelial dysfunction and fibrosis score. A significant endothelial dysfunction was found in patients with NAFLD, compared with control subjects. Our data suggest that the severity of hepatic steatosis, widely encountered in NAFLD patients, may predict endothelial dysfunction. The degree of fibrosis scores did not show any effect on endothelial dysfunction. The steatosis and fibrosis pathways are believed to occur through different mechanisms. Follow-up studies are necessary to determine to what extent this association affects long-term morbidity and mortality.
